# Retinal peripapillary nerve fiber and retinal ganglion cell layer thickening preceed atrophy in children and teenagers with optic disc drusen

**DOI:** 10.1038/s41598-025-25161-7

**Published:** 2025-11-07

**Authors:** L. Rudolph, V. Baldassarre, K. Halfter, C. Lottspeich, G. Rudolph, C. S. Priglinger

**Affiliations:** 1 Department of Ophthalmology, LMU University Hospital, LMU Munich, Mathildenstraße 8, 80336 Munich, Germany; 2 Institute of Medical Information Processing, Biometry and Epidemiology, Faculty of Medicine, Ludwig-Maximilians-University (LMU), Marchioninistr. 15, 81377 Munich, Germany; 3 Department of Medicine IV, LMU University Hospital, LMU Munich, Ziemssenstr. 5, 80336 Munich, Germany

**Keywords:** Optic disc drusen, RNFL, rGCL, Papilledema, Optic atrophy, Axonal congestion, Pediatric patients, Diseases, Medical research, Neurology, Neuroscience

## Abstract

In adults, optic disc drusen (ODD) are linked to thinning of the retinal ganglion cell layer (rGCL) and peripapillary retinal nerve fibre layer (pRNFL), but stage-specific pediatric changes remain unclear. This prospective cross-sectional study included 31 patients (61 eyes) with ODD and 21 age-matched controls (42 eyes), aged 6–18 years. pRNFL thickness was measured with Spectralis SD-OCT at 3.5 mm and 4.7 mm peripapillary rings; rGCL was assessed at the posterior pole. ODD were graded by OCT and ultrasound criteria as follows: A; deep ODD detectable by ultrasound, exclusively. B; deep ODD detectable by OCT and located below Bruch´s membrane (BM), C; superficial ≤ 5, and D; superficial > 5 ODD above BM, respectively. At 3.5 mm, stage D ODD showed significant pRNFL thinning in the temporal superior (TS), nasal superior (NS) and nasal (N) sector, while stage C showed significant thickening TS, NS, temporal inferior (TI), and globally. At 4.7 mm, stage D ODD showed thinning in all sectors, most pronounced TS, NS and TI; the temporal sector was most preserved. Stage C did not differ from controls here. rGCL was thickened in stage B superior (S), NS, nasal inferior (NS) and globally, and in stage C in all sectors but TI. Stage D showed significant global thinning in all sectors, most temporal, least nasal. Thus, rGCL thickening preceded pRNFL changes; 3.5 mm detected early congestion, 4.7 mm revealed atrophy; the papillomacular bundle was relatively preserved. In pediatric patients deep drusen and those advancing toward the optic nerve head surface first cause sector-specific swelling of both pRNFL and rGCL followed by atrophy in the very same sectors. Larger patient cohorts and prospective longitudinal studies are needed to define whether these findings occur in a chronological order. If so, such data may become of prognostic value for visual functions in ODD patients.

**Supplementary Information:**

The online version contains supplementary material available at 10.1038/s41598-025-25161-7.

## Introduction

Optic disc drusen (ODD) are acellular, calcified, hyaline deposits located in the optic nerve head with a prevalence of about 0.2% − 2.4% of the adult population^1,2^. Using OCT diagnostics the ”Copenhagen Child Cohort 2000 Eye Study” revealed a frequency of 1% at the age from 11 to 12 years^3^. Based on their location, ODD may be subdivided into visible ODD, seen on ophthalmoscopy and buried ODD, which are not disernible on fundoscopy due to their location deep within the optic nerve head^4^. Nevertheless, a clear definition of superficial/deep ODD has not been established.

Optic disc drusen seem to change their localisation and size throughout a person’s lifetime. In children ODD are mostly buried deep in the optic nerve head causing optic disc swelling. Clinically, distinguishing pseudopapilloedema due to optic disc drusen from other causes of papilledema in children can be challenging. In a retrospective case series Hoover et al.^5^ followed 40 children with pseudopapilledema and noted discrete hyaline bodies or papillary calcifications in one or both eyes that became visible at a mean age of 12.1 years. Similarly, Frisen and Malmqvist^6,7^ reported that the anatomically visible changes mainly take place during the teenager years and that ODD assumed a more superficial location during early adulthood^6^. In agreement with this, using EDI-OCT a recent study evidenced an actual displacement of drusen towards the surface of the optic nerve head in 67.4% of buried ODD in a young population^8^.

Histologically, ODD can be located at all levels of the prelaminar optic nerve, while the smaller ones tend to be closer to the lamina cribrosa^2^. Anterior to the ODD distended and vacuolated axons with large mitochondria are found suggestive of impaired axonal transport^9^. An axoplasmic stasis is further suggested by electron microscopy of the distened fibres revealing distressed mitochondria with needles of crystalline calcium. When the engorged optic nerve fibres bulge over the rim of the Bruch´s membrane opening (BMO) ODD-associated PHOMS arise as a structural correlate in SD-OCT, while the collective distension of the axons causes a visible elevation of the optic disc, clinically resembling papilledema^1,9,10^. As evidenced in a recent histopathological study thickening of the superficial RNFL was present in eyes with large ODD located in the BMO and the more superficial the ODD became, the thinner was the RNFL^1^. This is in line with recent studies using OCT, suggesting that large deep ODD might cause crowding of axons in the ONH leading to thickened pRNFL, pseudopapilledema and PHOMS, a finding found moustly in younger patients. Once optic disc drusen are located more along the surface, the thickness of the retinal nerve fibre layer decreases and PHOMS disappear^11–14^.

Recent electrophysiological studies revealed that axoplasmic stasis in ODD may not only affect peripapillary nerve fibers, but also the corresponding retinal ganglion cell somata and GCL function^15,16^. As evidenced in a cohort of 19 ODD eyes the 15´ pattern electroretinography (PERG) amplitude was reduced in 68% of eyes with superficial ODD and in only 16% of those with deep ODD. These PERG abnormalities in superficial ODDs significantly correlated with the visible ODD height and reduced pRNFL thickness and were ascribed to a functional rGCL impairment secondary to a local disturbance of axoplasmic transport at the optic disc. The authors suggested that the mechanical compression of the prelaminar RNFL caused by ODD may lead to a retrograde distension of the rGCL body and subsequent rGCL dysfunction^15–17^. In line with this rGCL soma swelling and dysfunction are a common finding in animal models of ONH injury, both as a sign of threatening cell death but also as a compensatory mechanism^18^. Thus, macular rGCL analysis might be useful for detecting early structural degeneration particularly in ODD, because ODD often produce pRNFL thickening that can mask axonal damage yielding a false negative result. In order to determine early structural changes associated with ODD we assessed structural changes at both, the rGCL and pRNFL level, as measured by SD-OCT in a pediatric and teenage cohort of patients with different stages of ODD. The data provided here show for the first time that rGCL swelling preceeds pRNFL atrophy as ODD become more superficial and that pRNFL atrophy mainly occurs in the very same sectors that were thickened in earlier stages.

## Methods

### Study design

The study was designed as a prospective, single-center observational, crossectional study.

### Study participants

A convenience sample of study participants with drusen was recruited from the University Eye Hospital of the Ludwig-Maximilians-University Munich. Healthy controls were recruited to approximately match the drusen cohort in both age and sex distribution. Due to the lack of previous data on child and teenage drusen patients an initial hypothesis regarding the study outcome was not formulated, rather the intent of the study was exploratory. Inclusion criterion was positive evidence of drusen in the ultrasound and/or OCT examination of the optic nerve head in the drusen cohort and the lack of such criteria in the control group. Exclusion criteria for both groups were any ocular or neurological disorders or any medication that could affect the visual system. Refraction was not allowed to exceed 6 dioptres of hyperopia or myopia and 4 dioptres of astigmatism. Best-corrected visual acuity (BCVA) had to be at least 0.8 decimal for each eye in normal subjects and was converted into the logMAR. Intraocular pressure had to be between 6 and 21 mmHg for both groups.

###  Ophthalmological assessment

All probands underwent BCVA-testing, IOD-measurement, slit-lamp examination and dilated funduscopy, Goldmann visual field testing, SD-OCT and ultrasound for exclusion or verification of drusen, respectively. A signal increase at 40 db or below in the area of the optic nerve head was classified as drusen. The optic nerve heads of all subjects were assessed by the Spectralis™SD-OCT (Heidelberg Engineering, Heidelberg, Germany) on each eye. We obtained a 15 × 10 degree rectangle horizontal raster scan centred on the optic disc with 97 Sect. (30 μm between each scan, each section had 20 frames averaged). The scan of each eye was repeated thereafter in Spectralis enhanced depth imaging (EDI-OCT) mode to maximize the chance of detecting buried drusen. pRNFL was measured using the optic disk circle protocols in 3.5 mm and 4.7 mm diameter around the optic nerve head. Posterior pole algorithm of the Spectralis™SD-OCT was performed to measure the retinal ganglion cell layer (rGCL) thickness at the posterior pole using the device´s automatic segmentation. The 8 × 8 PPA scans (61 cross-sectional images at a distance of 120 μm) generate a macular cube measuring 30° × 25° centred on the fovea and oriented using a fovea-disk alignment. Two different forms of presentation were chosen to visualise the results. First an elliptical ring that has an inner radius of 0.618 mm in the horizontal axis and 0.531 mm in the vertical axis and an outer radius of 1.857 mm horizontally and 1.590 mm vertically. It is divided into 6 sectors with angles of 60°. These 6 sectors of the macular grid correspond to the superior (S), inferior (I), temporal-superior (TS), temporal-inferior (TI), nasal-superior (NS), and nasal-inferior (NI) macular region. The grid is normalized to the axis between the centre of Bruch’s membrane opening of the disc and the foveola by Heidelberg`s Anatomic Positioning System. Second a macular grid measuring 24° × 24°, that is divided into 64 cells, each measuring 3° × 3°, which are distributed in eight rows and eight columns (8 × 8) was used^19^. To compare the rGCL thickness between the different groups and sectors, the measured values of the elliptical grid were chosen, the values for the rectangular grid are only shown in the supplementary figures (Suppl. Fig. S1) to demonstrate the comparability of our data to the data of Palazon-Cabanes et al.^19^. Scans to detect optic disc drusen were performed according to the recommendations of the Optic Disc Drusen Studies Consortium^4^. The OCT scans of the optic nerve heads of drusen patients were evaluated by 2 independent observers and assigned to the different subgroups. In cases of doubt, agreement was reached following a joint assessment. All ODD patients, who reported symptoms indicating increased intracranial pressure, were evaluated by cMRI and lumbar puncture if necessary for exclusion of an underlying intracranial pathology.

### Classification system of ODD

**Fig. 1:**
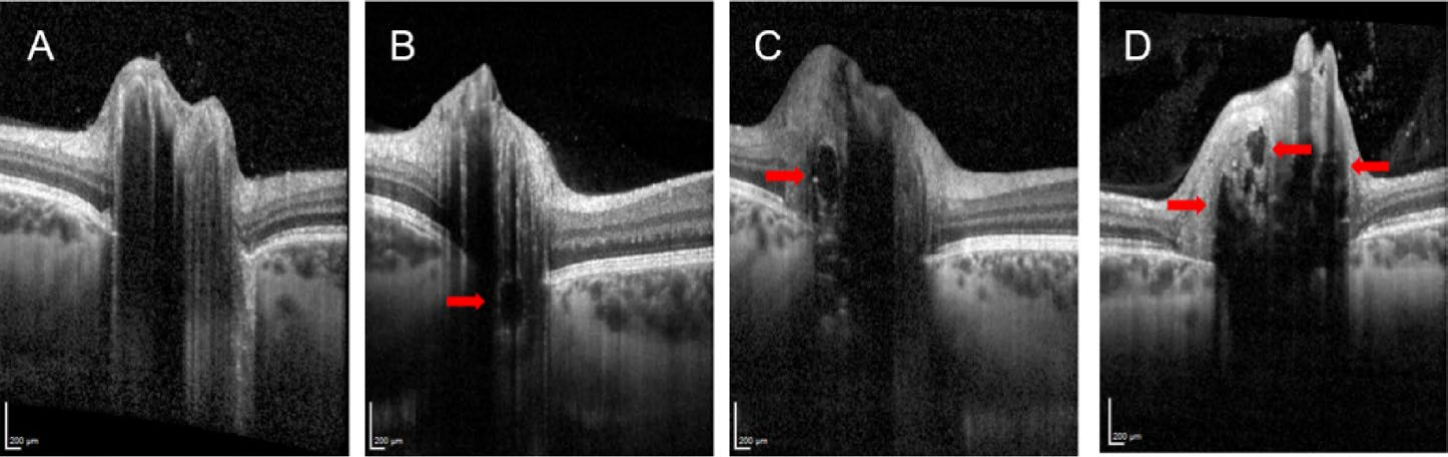
Representative samples for the OCT-based the classification system for ODD as described in the materials and methods section. Group A: deeply located drusen, detectable by ultrasound, exclusively (**A**). Group B: ODD discernible by OCT but still located below the level of Bruch´s membrane (**B**). Group C: single drusen (1–5) above the level of the Bruch´s membrane, regardless of whether deep drusen are also present (**C**). Group D: more than five ODD discernable by OCT above the level of Bruch membrane (**D**). The red arrow depicts ODD.

pRNFL and rGCL thickness of the whole cohort of patients with optic disc drusen showed a high variability (SD global pRNFL 3.5 mm circle: patients = 31.88 μm, normal controls = 8.82 μm; global pRNFL 4.7 mm circle: patients = 16.63 μm, normal controls = 6.35 μm, global rGCL: patients = 8.76 μm, normal controls = 3.20 μm, see Table 2). For this reason patients were divided into four subgroups as follows: Group A comprised patients with deeply located drusen, which were detectable in the ultrasound, exclusively. Patients whose drusen were also detected by OCT but still located below the level of Bruch´s membrane formed group B. Group C consisted of patients who showed single drusen (1–5) above the level of the Bruch´s membrane, regardless of whether deep drusen were also present. The patients in group D showed more than five drusen discernable by OCT, in some cases also confluent drusen above the level of Bruch´s membrane. Representative OCT-images are presented in Fig. 1.

### Statistical analysis

Statistical analysis was performed using IBM SPSS Statistics Version 29.0.1.0. The Mann-Whitney U test was used to consider whether pRNFL and rGCL thickness between two groups showed significant differences. For comparison between more than two groups the Kruskal-Wallis test was used. Fishers Exact test was used to compare categorial data. A probability p value of less than 0.05 was considered to be significant. Data are presented as mean ± SD and range (minimum - maximum). We reported the results both, for all eyes and patients, respectively and describe both eyes of our patients individually.

All eyes of ODD patients were considered individually and all affected eyes were included, based on our clinical opinion that excluding one eye of each patient or using the mean of both eyes, would have excluded relevant information on changes in the rGCL and pRNFL that have previously not been reported. Nevertheless, we did assess dependence by conducting a pearson correlation between both eyes for the central results. We also conducted a sensitivity analysis only considering left and right eyes individually.

All participants or their legal representatives were provided informed consent during the study. This prospective study complied with the tenets of the Declaration of Helsinki and was approved by the Ethics Committee of the Faculty of Medicine of Ludwig-Maximillians-University Munich (#19–911).

## Results

### Patient demographics

A total of 53 probands were included in the study (103 eyes). These comprised 32 pediatric patients and adolescents with optic disc drusen (61 eyes: 30 right eyes, 31 left eyes; *n* = 29 binocular drusen, *n* = 3 monocular drusen; *n* = 9 male, *n* = 23 female) and 21 healthy age-matched controls (42 eyes, *n* = 7 male, *n* = 14 female). Applying the OCT criteria as described in the Methods Sect. 10 eyes were assigned to group A, 12 to group B, 19 to group C and 20 to group D. A total of 3 of the 32 patients with drusen had unilateral findings and 14 of the 29 patients with bilateral findings had both eyes classified into different subgroups.

Representative examples for the OCT-based classification are illustrated in Fig. 1.

Looking at 29 patients who showed bilateral drusen there was a significant positive correlation between both eyes for global rGCL (pearson correlation 0.927, *p* < 0.001), for the global pRNFL in the 3.5 mm ring (pearson correlation 0.786, *p* < 0.001) and for the 4.7 mm ring (pearson correlation 0.852, *p* < 0.001). A sensitivity analysis, carried out separately for the right and left eyes, showed that the effects for the central results rGCL global and pRNFL global for the 3.5 and for the 4.7 mm ring were robust (see Table S1).

Mean age between the different subgroups of drusen patients differed significantly (*p* = 0.023, Kruskal-Wallis-Test). Patients whose eyes showed visible and more superficial drusen on OCT (groups B-D) were significantly older than those patients in whom the drusen were only visible by ultrasound (B-D: mean: 12.88y, range: 8-18y, SD: 2.56y; A: Mean: 10.30y, range: 6-16y, SD: 3.09y; *p* = 0.017). As subgroups A, B, and C of the drusen patients have different ages an effect of age on rGCL and pRNFL thickness was ruled out for the control cohort. Correlating age and global rGCL thickness (Pearson) yields a weak negative correlation of -0.022 (the younger the subject, the thicker the rGCL), which was not significant with *p* = 0.892. Correlating age and global pRNFL thickness for the 3.5 mm ring shows no correlation (Pearson correlation = 0.00, *p* = 1.00), for the 4.7 mm ring a positive not significant correlation was shown (pearson correlation coefficient = 0.075, *p* = 0.635).

Patient demographics are given in Table 1. Age-dependence of ODD location is illustrated in Suppl. Fig. S2.

**Table 1:** Patient demographics.

	ODD	Control	*p*-Value
Individuals (n)	32	21	
Number of eyes (n)	61 RE *n* = 30, LE *n* = 31	42 RE *n* = 21, LE *n* = 21	
Gender: n (%)	m: 9 (28%) f: 23 (72%)	m: 7 (33%) f: 14 (67%)	0.764 *
Age: mean, +/-SD, range [years]	12.44 (± 2.8), 6–18	12.24 (± 2.7), 8–17	0.769 **
Refractive error: mean SE, (+/-SD), range [dpt]	+ 0.25 (± 2.17) -6.0 to + 6.0	+ 0.24 (± 1.98) -4.0 to + 6.0	0.470 **
BCVA (logMAR) mean, (+/-SD), range	-0.04 (± 0.06) -0.2 to 0.1	-0.06 (± 0.09) -0.2 to 0.1	0.316 **
ODD subgroups	n, eyes	Mean age, range [years]	0.023***
Group A (n)	10	10.30 (6–16)	
Group B (n)	12	12.67 (8–16)	
Group C (n)	19	12.05 (8–16)	
Group D (n)	20	13.80 (10–18)	

n, number of individuals/eyes; ODD, optic disc drusen; SD, standard deviation; SE, spherical equivalent, RE, right eye; LE, left eye; BCVA, best corrected visual acuity; * Fisher´s exact test; ** Mann Whitney test; *** Kruskal Wallis test.

## pRNFL thickness

### 3.5 mm circle

**Fig. 2:**
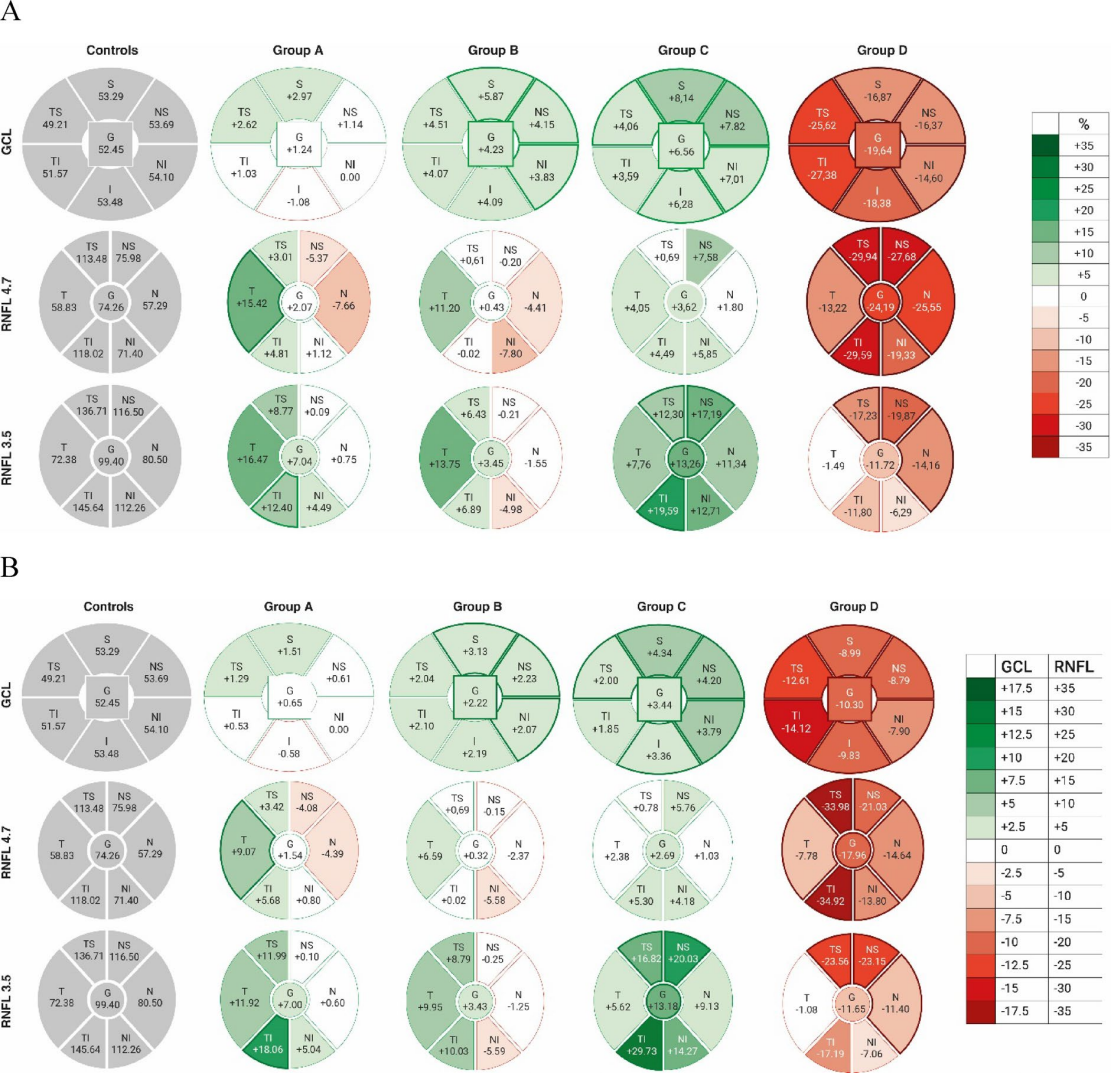
Graphical illustration of differences in GCL and RNFL thickness [A, in %; B in µm] between the ODD subgroups A, B, C and D and healthy controls as referred to in Table 3. Thickening versus control is illustrated in shades of green, thinning in red. Thickened outlines of a sector indicate a significant difference (*p* < 0.05, Mann Whitney Test). G (global), S (superior), I (inferior), N (nasal) und T (temporal), and respective combinations. Groups A, B, C and D indicate the subgroups of the drusen population.

At the the 3.5 mm circle subjects with ODD showed a trend towards global pRNFL thickening when compared to age-matched controls (ODD: 101.51 ± 31.89 μm, controls: 99.40 ± 8.82 μm). In the subgroup analyses this trend reached statistical significance for A in the temporal inferior (TI) (+ 18.06 μm/+12.40%, *p* = 0.046) and group C in the global (G) (+ 13.18 μm/+13.26%, *p* = 0.003), the temporal superior (TS) (+ 16.82 μm/+12.30%, *p* = 0.024), nasal superior (NS) (+ 20.03 μm/+17.19%, *p* = 0.026) and the TI sector (+ 29.73 μm/+19.59%, *p* < 0.001) when comparing to healthy controls. In clear contrast, in group D, comprising patients with the most superficial ODD, pRNFL thickness at the 3.5 mm circle was significantly lower than in normal subjects in the TS (-23.56 μm/-17.23%, *p* = 0.024), NS (-23.15 μm/19.87%, *p* = 0.004), and N sector (-11.40 μm/-14.16%, *p* = 0.003). As a consequence, comparing group C to group D a statistically significant RNFL loss was evident for the global (C: 112.58 vs. D: 87.75 μm; *p* = 0.010) as well as the TS (C: 153.53 vs. D: 113.15 μm; *p* = 0.013), NS (C: 136.53 vs. D: 93.35 μm; *p* = 0.005), TI (C: 175.37 vs. D: 128.45 μm; *p* = 0.014), N (C: 89.63 vs. D: 69.10 μm; *p* = 0.006) sector, suggesting that in progression from ODD stage C to D RNFL swelling converts into pRNFL loss in exactly those sectors that exhibited the most pronounced thickening in the earlier stages. The N pRNFL sector showed a more pronounced decrease in thickness than the T sector. This naso-temporal asymmetry was significant for group D when compared to controls (*p* = 0.034). Figure 2 graphically illustrates the percentage (A) and numeric (B) differences in pRNFL thickness in ODD subgroups A, B, C and D versus controls, respectively. Data are compiled in Tables 2 and 3.

**Table 2:** Peripapillary RNFL and retinal GCL thickness in patients with ODD and controls as determined by SD-OCT.

	Controls *n* = 42	ODD *n* = 61	Group A *n* = 10	Group B *n* = 12	Group C *n* = 19	Group D *n* = 20
RNFL 3.5 G	99.40 (8.82), 79–116	101.51 (31.88), 33–205	106.40 (23.41), 63–151	102.83 (9.85), 86–122	112.58 (16.64), 88–149	87.75 (47.92), 33–205
RNFL 3.5 T	72.38 (10.41), 53–93	77.69 (22.44), 36–133	84.30 (17.61), 58–108	82.33 (22.13), 61–126	78.00 (12.40), 61–101	71.30 (30.56), 36–133
RNFL 3.5 TS	136.71 (21.04), 92–182	137.92 (47.70), 30–256	148.70 (26.11), 88–184	145.50 (26.26), 106–193	153.53 (35.88), 58–211	113.15 (65.23), 30–256
RNFL 3.5 TI	145.64 (14.47), 121–179	154.20 (53.32), 36–282	163.70 (31.04), 113–216	155.67 (24.40), 133–220	175.37 (37.60), 92–243	128.45 (75.13), 36–282
RNFL 3.5 N	80.50 (11.53), 48–100	79.46 (34.20), 23–216	81.10 (30.86), 37–144	79.25 (14.30), 52–101	89.63 (24.94), 45–143	69.10 (48.14), 23–216
RNFL 3.5 NS	116.50 (24.14), 66–196	115.11 (49.36), 33–269	116.6 (37.54), 62–198	116.25 (16.67), 92–142	136.53 (43.82), 53–252	93.35 (63.93), 33–269
RNFL 3,5 NI	112.26 (22.24), 83–170	114.11 (42.78), 33–245	117.3 (25.04), 76–145	106.67 (20.42), 79–157	126.53 (33.78), 68–197	105.20 (62.29), 33–245
RNFL 4.7 G	74.26 (6.35), 63–86	69.52 (16.63), 27–99	75.80 (10.96), 63–95	74.58 (6.23), 64–85	76.95 (9.53), 59–95	56.30 (20.79), 27–99
RNFL 4.7 T	58.83 (8.56), 45–77	59.80 (13.67), 28–89	67.90 (12.75), 54–87	65.42 (12.94), 51–89	61.21 (8.55), 49–79	51.05 (14.49), 28–75
RNFL 4.7 TS	113.48 (14.60), 77–146	103.28 (30.31), 34–149	116.90 (17.42), 88–149	114.17 (13.97), 95–134	114.26 (22.36), 47–149	79.50 (35.82), 34–146
RNFL 4.7 TI	118.02 (13.51), 95–157	109.15 (32.54), 27–167	123.70 (14.73), 102–147	118.00 (12.95), 105–142	123.32 (21.57), 69–154	83.10 (40.33), 27–167
RNFL 4.7 N	57.29 (6.59), 44–73	51.62 (16.46), 15–92	52.90 (12.70), 37–73	54.92 (9.80), 37–69	58.32 (14.71), 32–89	42.65 (19.54), 15–92
RNFL 4.7 NS	75.98 (15.41), 45–130	70.18 (22.76), 27–121	71.90 (14.92), 51–99	75.83 (10.25), 61–92	81.74 (20.18), 46–118	54.95 (26.17), 27–121
RNFL 4.7 NI	71.40 (14.49), 47–110	67.21 (17.81), 28–108	72.20 (12.45), 52–87	65.83 (8.69), 52–87	75.58 (16.62), 48–108	57.60 (21.01), 28–98
GCL G	52.45 (3.20), 44–59	50.69 (8.76), 26–62	53.10 (2.60), 49–57	54.67 (3.94), 46–61	55.89 (3.21), 51–62	42.15 (10.17), 26–57
GCL TS	49.21 (3.93), 39–57	46.62 (9.95), 20–60	50.50 (2.42), 47–54	51.25 (4.20), 42–58	52.21 (3.65), 47–60	36.60 (11.35), 20–54
GCL TI	51.57 (3.85), 40–60	48.02 (11.03), 17–60	52.10 (3.60), 45–57	53.67 (4.19), 46–60	53.42 (3.87), 48–60	37.45 (13.33), 17–59
GCL S	53.29 (3.93), 39–60	52.56 (8.79), 28–67	54.80 (2.39), 52–60	56.42 (4.74), 49–67	57.63 (3.89), 52–66	44.30 (10.20), 28–57
GCL NS	53.69 (2.99), 47–60	52.66 (8.29), 31–63	54.30 (2.16), 52–57	55.92 (4.08), 46–63	57.89 (3.35), 53–63	44.90 (9.77), 31–59
GCL NI	54.10 (3.25), 47–62	53.10 (7.45), 33–63	54.10 (2.38), 51–58	56.17 (4.26), 46–62	57.89 (3.32), 53–63	46.20 (8.48), 33–59
GCL I	53.48 (3.02), 46–60	51.64 (8.77), 23–62	52.90 (2.96), 49–57	55.67 (3.80), 50–61	56.84 (3.67), 50–62	43.65 (10.60), 23–58

RNFL, peripapillary retinal nerve fiber layer; GCL, retinal ganglion cell layer; RNFL or GCL sectors: G (global), S (superior), I (inferior), N (nasal) und T (temporal), and respective combinations. Groups A, B, C and D indicate the subgroups of the drusen population. 3.5/ 4.7 mm diameter circle around the optic nerve head; Sectors: G (global), S (superior), I (inferior), N (nasal) und T (temporal) and respective combinations. Groups A, B, C and D indicate the subgroups of the drusen population; measurements are given in µm. SD in brackets, range below.

**Table 3:** Comparison of peripapillary RNFL thickness between ODD groups and controls at 3.5 mm diameter circle around the optic nerve head as determined by SD-OCT.

RNFL Sector	*p*-value ODD v. Contr.	*p*-value A v. Contr.	Diff. A v. Contr. [µm/%]	*p*-value B v. Contr.	Diff. B v. Contr. [µm/%]	*p*-value C v. Contr.	Diff. C v. Contr. [µm/%]	*p*-value D v. Contr.	Diff. D v. Contr. [µm/%]	*p*-value D v. C
RNFL 3.5 G	0.240	0.210	+ 7.00/ + 7.04%	0.343	+ 3.43/ + 3.45%	0.003**	+ 13.18/ + 13.26%	0.078	-11.65/ -11.72%	0.010**
RNFL 3.5 T	0.265	0.052	+ 11.92/ + 16.47%	0.354	+ 9.95/ + 13.75%	0.133	+ 5.62/ + 7.79%	0.378	-1.08/ -1.49%	0.152
RNFL 3.5 TS	0.485	0.095	+ 11.99/ + 8.77%	0.441	+ 8.79/ + 6.43%	0.024**	+ 16.82/ + 12.30%	0.024**	-23.56/ -17.23%	0.013**
RNFL 3.5 TI	0.099	0.046**	+ 18.06/ + 12.40%	0.328	+ 10.03/ + 6.89%	< 0.001**	+ 29.73/ + 19.59%	0.115	-17.19/ -11.80%	0.014**
RNFL 3.5 N	0.327	0.478	+ 0.60/ + 0.75%	0.723	-1.25/ -1.55%	0.125	+ 9.13/ + 11.34%	0.003**	-11.40/ -14.16%	0.006**
RNFL 3.5 NS	0.665	0.701	+ 0.10/ + 0.09%	1.0	-0.25/ -0.21%	0.026**	+ 20.03/ + 17.19%	0.004**	-23.15/ -19.87%	0.005**
RNFL 3.5 NI	0.812	0.450	+ 5.04/ + 4.49%	0.473	-5.59/ -4.98%	0.084	+ 14.27/ + 12.71%	0.268	-7.06/ -6.29%	0.106

RNFL, peripapillary retinal nerve fiber layer; RNFL sectors: G (global), S (superior), I (inferior), N (nasal) und T (temporal), and respective combinations. Groups A, B, C and D indicate the subgroups of the drusen population. P- values obtained by Mann Whitney Test among groups, ** statistically significant (*p* < 0.05).

### 4.7 mm circle

At the 4.7 mm circle mean global RNFL thickness of ODD subjects showed a trend towards thinning compared to healthy controls (ODD: 69.52 ± 16.63 μm; controls: 74.26 ± 6.35 μm, *p* = 0.233). This was only significant for the nasal sector (*p* = 0.022). In the subgroup analysis a significant thinning was evident for all sectors of group D. The average decrease was greatest TS (-33.98 μm/29.94%; *p* = 0.001), NS (-21.03 μm/27.68%; *p* < 0.001), and TI (-34.92 μm/29.59%; *p* < 0.001). The smallest loss was evident in the temporal pRNFL sector (-7.78 μm/13.22%; *p* = 0.040). Likewise, a statistically significant difference was also evident when comparing group D and C for all sectors. The subgroups with deeper drusen (groups A-B) exhibited both, a trend towards swelling of the temporal RNFL when compared to in controls in the temporal sectors (T: A: 67.90, B: 65.42, vs. Co: 58.83 μm), and a trend towards thinning of the nasal sectors (N: A: 52.90, B: 54.92, vs. Co: 57.92 μm). Thickening was significant in the temporal sector of group A (+ 9.07 μm/+15.42%; *p* < 0.039). For details and p-values see Tables 2 and 4, for graphical illustration Fig. 2.

**Table 4:** Comparison of peripapillary RNFL thickness between ODD groups and controls at 4.7 mm diameter circle around the optic nerve head as determined by SD-OCT.

RNFL Sector	*p*-value Drusen v Contr.	*p*-value A v. Contr.	Diff. A v. Contr. [µm/%]	*p*-value B v. Contr.	Diff. B v. Contr. [µm/%]	*p*-value C v. Contr.	Diff. C v. Contr. [µm/%]	*p*-value D v. Contr.	Diff. D v. Contr. [µm/%]	*p*-value D v C
RNFL 4.7 G	0.233	0.935	+ 1.54/ + 2.07%	0.917	+ 0.32/ + 0.43%	0.329	+ 2.69/ + 3.62%	< 0.001**	-17.96/ -24.19%	0.001**
RNFL 4.7 T	0.564	0.039**	+ 9.07/ + 15.42%	0.145	+ 6.59/ + 11.20%	0.326	+ 2.38/ + 4.05%	0.040**	-7.78/ -13.22%	0.024**
RNFL 4.7 TS	0.268	0.719	+ 3.42/ + 3.01%	0.950	+ 0.69/ + 0.61%	0.602	+ 0.78/ + 0.69%	0.001**	-33.98/ -29.94%	0.006**
RNFL 4.7 TI	0.508	0.201	+ 5.68/ + 4.81%	0.851	-0.02/ -0.02%	0.193	+ 5.30/ + 4.49%	< 0.001**	-34,92/ -29.59%	0.001**
RNFL 4.7 N	0.022**	0.218	-4.39/ -7.66%	0.525	-2.37/ -4.41%	0.821	+ 1.03/ + 1.80%	< 0.001**	-14.64/ -25.55%	0.002**
RNFL 4.7 NS	0.284	0.429	-4.08/ -5.37%	0.771	-0.15/ -0.20%	0.139	+ 5.76/ + 7.58%	< 0.001**	-21.03/ -27.68%	0.001**
RNFL 4.7 NI	0.347	0.609	+ 0.80/ + 1.12%	0.343	-5.58/ -7.80%	0.418	+ 4.18/ + 5.85%	0.011**	-13.80/ -19.33%	0.008**

RNFL, peripapillary retinal nerve fiber layer; RNFL sectors: G (global), S (superior), I (inferior), N (nasal) und T (temporal), and respective combinations. Groups A, B, C and D indicate the subgroups of the drusen population. P- values obtained by Mann Whitney Test among groups, ** statistically significant (*p* < 0.05).

## Retinal GCL thickness

For optic disc drusen groups A, B and C global rGCL thickness at the posterior pole was higher than in normal subjects (global rGCL: A: 53.10, B: 54.67, C: 55.89, Co: 52.45 μm.). This reached statistical significance in group B (+ 2.22 μm/+4.23%, *p* = 0.001) and C (+ 3.44 μm/+6.56%, *p* < 0.001). Looking at the individual rGCL sectors, thickening in group B was significant in the S (+ 3.13 μm/+5.87%, *p* = 0.036), NS (+ 4.20 μm/+7.82%, *p* = 0.015) and NI sector (+ 2.07 μm/+3.83%; *p* = 0.036) and in group C in all sectors (TS: +2.00 μm/ +4.06%, *p* = 0.009; S: +4.34 μm/ +8.14%, *p* < 0.001; NS: +4.20 μm/+7.82%, *p* < 0.001; NI: +3.79 μm/ +7.01%, *p* < 0.001, I: +3.36 μm/+6.28% *p* = 0.002) except TI. In contrast, in group D global rGCL (-10.30 μm/-19.64%, *p* < 0.001) and all individual sectors were significantly thinner than in normal controls, suggesting that, GCL soma swelling in patients with more buried drusen, may preceed rGCL atrophy that occurs when ODD accumulate above Bruch`s membrane in high amounts (Table 5; Fig. 2). Further analysis of the pattern of GCL loss in group D revealed that the nasal quadrants, representing the papillo-macular fibers displayed the smallest loss when compared to healthy controls (NS: -8.79 μm/-16.37%, NI: -7.90 μm/-14,60%). Intriguingly, rGCL consistently showed an asymmetry in thickness between the nasal and temporal hemifield for both controls and ODD patients being thicker nasally than temporally. The mean values of the NS and NI and those of the TS and TI sectors differed by 3.50 μm in controls, 2.90 μm in group A, 3.58 μm in group B, 5.08 μm in group C, and 8.53 μm in group D respectively. The naso-temporal difference was significantly higher in group D versus controls (*p* < 0,001). The decrease in rGCL thickness was more pronounced temporally than nasally (for graphical illustration see Fig. S3).

**Table 5:** Comparison of macular retinal GCL thickness between ODD groups at the posterior pole as determined by SD-OCT.

GCL Sector	*p*-value Drusen v. Contr.	*p*-value A v. Contr.	Diff. A v. Contr. [µm/%]	*p*-value B v. Contr.	Diff. B v. Contr. [µm/%]	*p*-value C v. Contr.	Diff. C v. Contr. [µm/%]	*p*-value D v. Contr.	Diff. D v. Contr. [µm/%]	*p*-value D v. C
GCL G	0.513	0.639	+ 0.65/ + 1.24%	0.040**	+ 2.22/ + 4.23%	< 0.001**	+ 3.44/ + 6.56%	< 0.001**	-10.30/ -19.64%	< 0.001**
GCL TS	0.856	0.233	+ 1.29/ + 2.62%	0.105	+ 2.04/ + 4.15%	0.009**	+ 2.00/ + 4.06%	< 0.001**	-12.61/ -25.62%	< 0.001**
GCL TI	0.586	0.608	+ 0.53/ + 1.03%	0.158	+ 2.10/ + 4.07%	0.135	+ 1.85/ + 3.59%	< 0.001**	-14.12/ -27.28%	< 0.001**
GCL S	0.284	0.380	+ 1.51/ + 2.97%	0.036**	+ 3.13/ + 5.87%	< 0.001**	+ 4.34/ + 8.14%	0.003**	-8.99/ -16.87%	< 0.001**
GCL NS	0.181	0.497	+ 0.61/ + 1.14%	0.015**	+ 2.23/ + 4.15%	< 0.001**	+ 4.20/ + 7.82%	0.002**	-8.79/ -16.37%	< 0.001**
GCL NI	0.484	0.991	+ 0.00/ + 0.00%	0.036**	+ 2.07/ + 3.83%	< 0.001**	+ 3.79/ + 7.01%	< 0.001**	-7.90/ -14.60%	< 0.001**
GCL I	0.885	0.575	-0.58/ -1.08%	0.088	+ 2.19/ + 4.09%	0.002**	+ 3.36/ + 6.28%	< 0.001**	-9.83/ -18.38%	< 0.001**

GCL, macular retinal ganglion cell layer thickness as determined by SD-OCT; differences between subgroups are presented as µm (top) and percentage (below). GCL sectors: G (global), S (superior), I (inferior), N (nasal) und T (temporal), and respective combinations. Groups A, B, C and D indicate the subgroups of the drusen population. P-values obtained by Mann Whitney Test among groups, ** statistically significant (*p* < 0.05).

## Discussion

In the present study we in a cohort of pediatric and teenage patients provide first evidence that pRNFL and rGCL atrophy in advanced, superficial drusen may be preceeded by subclinical axonal congestion and rGCL soma swelling in earlier stages. Using an OCT-based grading system for ODD rGCL thickening was evident before significant pRNFL swelling appeared. As ODD advanced towards the surface this was accompanied by pRNFL thickening at the 3.5 mm circle, which mainly occured in those sectors that were found to become atrophic once ODD have accumulated above Bruch´s membrane. When comparing the 3.5 and 4.7 pRNFL circles, there was a more pronounced swelling in group C at the 3.5 mm location, whereas atrophy in group D was most evident in the 4.7 mm circle. Intriguingly, the temporal pRNFL sector, which represents the papillo-macular bundle, showed the smallest swelling in group C and the smallest atrophy in group D. In line with this rGCL atrophy was less pronounced nasally.

In the present study we used OCT imaging combined with ultrasound of the ONH to classify subjects according to the number and location of drusen in the optic nerve head. Classifications of ODD in previous studies mainly relied on ophthalmoscopic and ultrasound-based criteria^11,14^, which do not allow for further subclassification of those ODD that are not visible at the surface. For these reasons we chose to grade ODD into two stages beyond (A, B) and two stages above Bruch´s membrane (C, D), respectively. As suggested by Malmqvist et al. Bruch’s membrane (BM) is a well-defined structure in OCT and appeared appropriate as an anatomical demarcation for their localisation^20^. Since pRNFL thickness differed between patients with a few drusen above BM and those whose ONH was packed with superficial drusen we chose to define less than 5 drusen above BM as a cut-off for group C and D. The stratification to group A arose from the finding that our cohort included eyes where ODD were not detectable by EDI-OCT, but clearly by the ultrasound criteria applied. Group B was defined as those ODD that were still underneath Bruch´s membrane, but clearly detectable by OCT. The definition of group A seems somewhat contradictory in the light of the study by Merchant et al., who demonstrated the superiority of EDI-OCT over ultrasound for detecting deep drusen^21^. One explanation could be that the patients in our cohort are young and may have deeper and less calcified drusen than the mainly adult patients in Merchant’s cohort. Also, the ultrasound criteria were slightly different in the two studies.

Patients in group A were the youngest, followed by group B and C, and patients in group D were the oldest individuals of our ODD cohort. This corresponds to the findings in the literature, which assume an increase in drusen over time and a movement towards the surface of the optic disc^5–8,22^.

Looking at the mean pRNFL values of the whole ODD cohort the 3.5 mm circle suggested a trend towards pRNFL thickening, but without significance and with large standard deviations. Only when applying OCT-criteria of different grades of ODD we were able to delineate a significant decrease of pRNFL thickness in the TS, NS, and N sector in group D, representing most advanced, superficial ODD. In contrast, in group C, defined as less superficial drusen, pRNFL was significantly thickened in almost the very same sectors, namely TS, NS, TI. The phenomenon of peripapillary atrophy in mainly superior and inferior sectors in advanced and RNFL swelling in earlier ODD stages has been described by several authors^14,17,23–25^, especially those who examined younger patients. For example, Alarcon et al. found the global pRNFL to be significantly thinner in young patients with superficial ODD, whereas global and inferior pRNFL were significantly thicker in buried drusen, somewhat comparable to our findings in group C. As in our study, these differences were not significant without ODD subclassification^23^. This underscores the necessity to subclassify ODD in order to monitor their impact on pRNFL thickness. Also, if the respective changes caused by different stages of drusen (our groups A-D) are brought into a chronological context, the relatively short-term fluctuations of the pRNFL observed in ODD patients younger than 20 years, but not in patients older than 20 years, can be explained^26^.

At the 4.7 mm circle findings in group D were comparable to those from the 3.5 mm ring, i.e. pRNFL was significantly reduced in all sectors. However, in Group C, comprising subjects with less superfical drusen than group D, at the 4.7 mm location unlike to the 3.5 mm ring where the pRNFL was significantly thickened in the TS, NS, and TI sectors, only a trend toward thickening in the NS, TI, and NI sectors was observed. Interestingly, in group D, the relative pRNFL loss at 4.7 mm was even greater than at the 3.5 mm location and more pronounced in TS, TI, and NS, when compared to the T sector. Therefore, measurements of pRNFL at the 4.7 ring may enable to earlier recognize damage to the pRNFL in advancing drusen than at the 3.5 mm location, where due to the proxmity to the optic nerve head pRNFL damage may still be masked by axonal congestion. Vice versa the 3.5 ring, again due to its proximity to the optic nerve head, may more clearly show the sectors of congestion and indicate those at highest risk for later atrophy. This assumption is based on the finding that at the 3.5 mm ring pRNFL thinning in group D is found in the very same sectors that exhibit thickening in group C. Of interest, in group D pRNFL loss was more pronounced NS, TS and TI when compared to the T sector, suggesting that the temporal sector, containing fibres of the papillo-macular bundle, was the most preserved at both locations. This fits well into the context of a primary nasally located damage to the pRNFL in the ONH, as suggested by the study of Nana Wandji et al.^27^ who spotted buried optic disc drusen mainly in the nasal region of the optic nerve head and found damaged pRNFL predominantly in the superior and nasal sectors.

From these data pRNFL measurements both, at the 3.5 and 4.7 mm ring, have strengths and limitations when assessing swelling and atrophy of the pRNFL in ODD. Our findings underscore the value of a combined assessment of the two locations in prominent optic nerve heads. Unfortunately, with a few exceptions little consideration has been given this issue in studies examining ODD^28^.

Interestingly, the asymmetry of pRNFL thickness continued into the rGCL, which consistently was thicker nasally than temporally in all groups. The nasal-temporal rGCL asymmetry can also be found in healthy eyes^19,29^, but in the present ODD cohort the extent of asymmetry increased with advancing ODD stage and became significantly different when compared to controls in group D. This fits well to our hypothesis, that the papillo-macular bundle represented by temporal pRNFL and nasal rGCL sectors is relatively preserved in advanced ODD stages, while the superior and inferior arcuate pRNFL fibers, which originate from the temporal rGCL hemifield, are more affected by ODD-induced damage. This relative preservation of fibers and GCL of the papillo-macular bundle may help explain the preservation of central visual acuity, which is often seen in drusen patients showing advanced visual field defects^12,14^. Of interest, an asymmetry in rGCL thickness in ODD and papilledema was also noticed by Pilat et al.^30^. Their data show a thinner rGCL temporally than nasally, which was not significant in ODD, but in true papilledema. Unfortunately this phenomenon was not further discussed in the article.

rGCL thinning in ODD as found in our patients with superficial drusen is not a novel finding^15,23,24,28,30^. However, an rGCL thickening in subjects with deep ODD that preeceds atrophy has not been described before. If we consider the subgroups as different stages of drusen a first significant rGCL thickening of several neighbouring sectors is evident in group B in the S, NS and NI sector, whereas pRNFL at that stage was neither significantly thickened at the 3.5 nor at the 4.7 mm ring. Only in group C, when the rGCL already showed significant swelling in almost all sectors, this was paralleled by corresponding pRNFL thickening of several neighbouring sectors at the 3.5 mm circle. To the best of our knowledge this phenomenon has uniquely been described by Pilat at al.^30^. They reported a significantly thicker rGCL in drusen patients at the posterior pole compared with controls, which they did not conclusively comment. When assuming a chronological sequence of the different subgroups these data suggest that rGCL swelling preceeds atrophy and occurs at even earlier ODD stages than pRNFL thickening. Conversly, in the ODD stages as defined here, rGCL thinning does not preceed atrophy of the pRNFL and from our data can therefore not be used as an early indicator for pending atrophy. Nevertheless, it must be noted that the data provided here are crossectional and do not contain longitudinal information. Prospective, longitudinal studies are warranted to finally confirm the assumption that the subgroups described represent temporally successive stages of drusen development.

Taken togehter data from this study fit harmoniously with the concept of an axoplasmatic stasis in ODD pathogenesis. Moreover, we provide novel insights into the changes of rGCL and pRNFL thickness in different stages of ODD. In this concept an axoplasic stasis acts as a possible trigger factor for progression of drusen formation, consecutive pRNFL congestion and lateron atrophy. Many studies, especially those that have included adult patients, provide information on the areas of the optic nerve head, where the greatest damage to the pRNFL occurs. For example, Engelke et al. in adults with ODD reported a reduction of the pRNFL, especially in the superior hemifield compared to normal controls^28^, and Teixeira et al. found a significant positive association between optic disc drusen and decreased pRNFL thickness in the nasal, superonasal and inferotemporal sectors in children under eighteen^31^. Interestingly, in most studies including our own, superior and inferior sectors are affected first, which resembles the pattern found in optic neuropathies such as glaucoma and papilledema, both of which are attributed to a translaminar pressure gradient, although in glaucoma in the opposite, posterior direction^14,32^. In line with the pattern of pRNFL damage, arcuate field defects, nasal steps and constricted fields in papilledma and ODD are strongly reminiscent of findings in glaucoma, thought to be caused by a mechanical strain at the lamina cribrosa^14,32^. The hypothesis of a common pathogenesis of true papilledema and ODD is further supported by histological studies^9^. At the cellular level increased mitochondria in both, ODD and papilledema, are suggestive of impaired axonal transport and axoplasmic stasis, leading to mechanical compression of nerve fibers^9^. The reasons leading to axoplasmatic stasis in ODD remain elusive and many theories have been proposed. In most hypotheses the lamina cribrosa plays a critical role as a chokepoint for the stasis of axoplasmic transport. Narrow scleral canals that are found in the majority of ODD patients further support the hypothesis of an axonal congestion^1,33^. In this context, the progressive rGCL layer thickening in group B and C, before atrophy occurs is a major finding from this study. Again, there may be paralells to true papilledema associated with intracranial hypertension. The “idiopatic intracranial hypertension (IIH) treatment trial” also found a mild correlation between rGCL + IPL thickness and RNFL-thickness in patients with true papilledema^34^. The authors concluded that the axonal stasis associated with papilledema can affect both axons and soma of retinal ganglion cells in the macula. In addition, they pointed out that, if this does occur, early thinning of the GCL + IPL-associated neuronal loss in IIH may be more difficult to detect. Of note, in severe papilledema caused by IIH an incorrect segmentation of the retinal layers at the edge of prominent optic nerve heads could be a possible source of error that leads to a rGCL that is falsely assumed to be thickened^35^. These segmentation problems are not relevant for the rGCL thickness used here for comparison between different groups, as the calculated elliptical area did not extend to the margin of the optic disc.

A major limitation of this study is its crossectional nature, the relatively small and non-homogeneous number of patients in the different subgroups and the fact that the criteria for classification into the different subgroups were not identical to previous studies. Also the considerably higher standard deviation of the drusen patients compared to the normal subjects, which remained high even after subdivision into subgroups, especially in group D, limits the reliability of the data. Like some authors of previous studies, we considered the different subgroups of drusen patients as showing different degrees of alterations caused by drusen and placed them in a temporal context, even though our study does not contain longitudinal, but only cross-sectional data. Unfortunately, the relatively small number of studies dealing with drusen in childhood and adolescence exclusively limits comparability. Further, as our data are not longitudinal, no conclusions can yet be drawn on the visual outcome of the patients. Nevertheless, once longitudinal data of larger patient cohorts are available, data as provided here could become of prognostic value. Another limitation of this study is that the data were analyzed only in aggregate. The individual outcome measures were not compared within each subject, so it remains unclear whether the observed changes in corresponding anatomical regions also occur at the individual level. This clearly should be addressed in further analyses.

A strength of this study is that we combined analysis of 3.5 mm and 4.7 mm pRNFL and rGCL in order to limit a bias caused by swelling of the pRNFL that may mask an early axonal damage in drusen patients^17,24^. Using this approach we show here that each OCT parameter has its individual stage-dependent course, which does not necessarily parallel the trajectories of the other parameters, although they all follow the pattern of thickening followed by atrophy. The findings for each individual OCT parameter may be placed in a chronological context of ODD development – again, provided that the ODD stages proposed here are considered stages of the disease. We therefore propose that the combined use of the three parameters may be advisable in patients with ODD.

Furthermore, we applied a classification system of ODD that allowed for detection of stage-dependent differences, which were not evident when averaging pRNFL and rGCL data without prior subclassification. In the recent years the rapid development of OCT imaging has opened new avenues for subclassifying ODD, which may be particularly useful when examining subjects with deep ODD. However, it was not the intention of the present study to develop a new classification system or to recommend it to other study groups or to present it as superior to existing systems. Nevertheless, the classification system proposed here proved to be useful in interpreting OCT data in a pediatric/adolescent ODD cohort, where deep ODD are a common finding, and enabled to distinguish different degrees of deep ODD by OCT criteria. These findings suggest that ODD classification systems that integrate OCT-criteria may be superior to purely visual or ultrasound-based methods and should be taken in account in future classifications. From a clinical perspective assigning young drusen patients to subgroups seems useful as this can influence the frequency of clinical follow-up. For example, patients assigned to subgroups C and D may be monitored more frequently including visual field testing, as a floor effect in OCT in stage D may mask further deterioration. Since swelling converts to atrophy between stages C and D, it would be desirable to subclassify these two stages further to define more precisely the stage at which the risk of developing atrophy increases.

Finally, if the subgroups of ODD as proposed here are considered as different stages of ODD and are put in a chronological context, dynamic and stage-dependent changes in rGCL and pRNFL may occur. Advanced, superficial drusen (group D), are characterized by atrophy of the pRNFL and corresponding rGCL sectors. pRNFL atrophy is more clearly evident at the 4.7 mm than at the 3.5 mm pRNFL circle, most likely due to residual peripapillary axonal congestion in proximity of the optic nerve head. Conversely, pRNFL thickening occuring before atrophy can most clearly be seen in the 3.5 mm circle of group C and mainly occurs in those sectors of the 3.5 mm circle that exhibit the strongest atrophy in group D. Most importantly, rGCL shows significant swelling in even earlier stages (group B). rGCL cell atrophy is more pronounced in the temporal than in the nasal rGCL hemifield, and the corresponding temporal pRNFL, representing the papillo-macular bundle is best preserved in all ODD groups. Although not investigated in this study and speculative at this time, the phenomenon of swelling of the retinal nerve fibres and rGCL before atrophy adds to the hypothesis that the pattern of damage that occurs in advanced stages of ODD underlies a local pathophysiology caused by axoplasmic stasis or a translaminar pressure gradient. Literature in this respect is scarce and further prospective, longitudinal studies with larger patient cohorts clearly are warranted. These studies may open an avenue to identify markers of prognostic value for the visual functions in ODD patients and proof the temporal relationship of the different stages of ODD as proposed here. Future research in this respect would benefit from a consensus on an ODD classification system to assure comparability of the results together with the combined assessment of available parameters.

## Supplementary Information

Below is the link to the electronic supplementary material.

Supplementary Material 1

Supplementary Material 2

Supplementary Material 3

Supplementary Material 4

## Data Availability

Data are contained within the article and Supplemantary Materials.
