# Supplementary material for: Retinal peripapillary nerve fiber and retinal ganglion cell layer thickening preceed atrophy in children and teenagers with optic disc drusen
Source: Sci Rep. 2025 Nov 7;15:39001. doi: 10.1038/s41598-025-25161-7 (PMC12595069; doi:10.1038/s41598-025-25161-7)
Supplement: Supplementary file 1 — Supplementary Material 1 [file 41598_2025_25161_MOESM1_ESM.pdf]

Figure S1: rGCL thickness in controls determined by SD-OCT

**8**	21	23	24	26	27	25	23	22
**7**	23	27	31	35	36	31	26	24
**6**	25	32	42	51	51	43	33	26
**5**	24	33	49	40	41	56	41	28
**4**	28	38	52	42	40	57	42	30
**3**	27	34	43	51	51	44	33	25
**2**	23	26	30	33	33	29	24	22
**1**	22	23	24	24	24	23	22	22
	**1**	**2**	**3**	**4**	**5**	**6**	**7**	**8**

rGCL thickness [µm]

S1: rGCL thickness (rounded to whole numbers) at the posterior pole in normal subjects according to the presentation of Palazon-Cabanes. Numbers at the x and y axis ascend from temporal to nasal, and from inferior to superior, respectively [19].
