# Supplementary material for: Retinal peripapillary nerve fiber and retinal ganglion cell layer thickening preceed atrophy in children and teenagers with optic disc drusen
Source: Sci Rep. 2025 Nov 7;15:39001. doi: 10.1038/s41598-025-25161-7 (PMC12595069; doi:10.1038/s41598-025-25161-7)
Supplement: Supplementary file 2 — Supplementary Material 2 [file 41598_2025_25161_MOESM2_ESM.pdf]

Figure S2: Age-dependence of ODD location

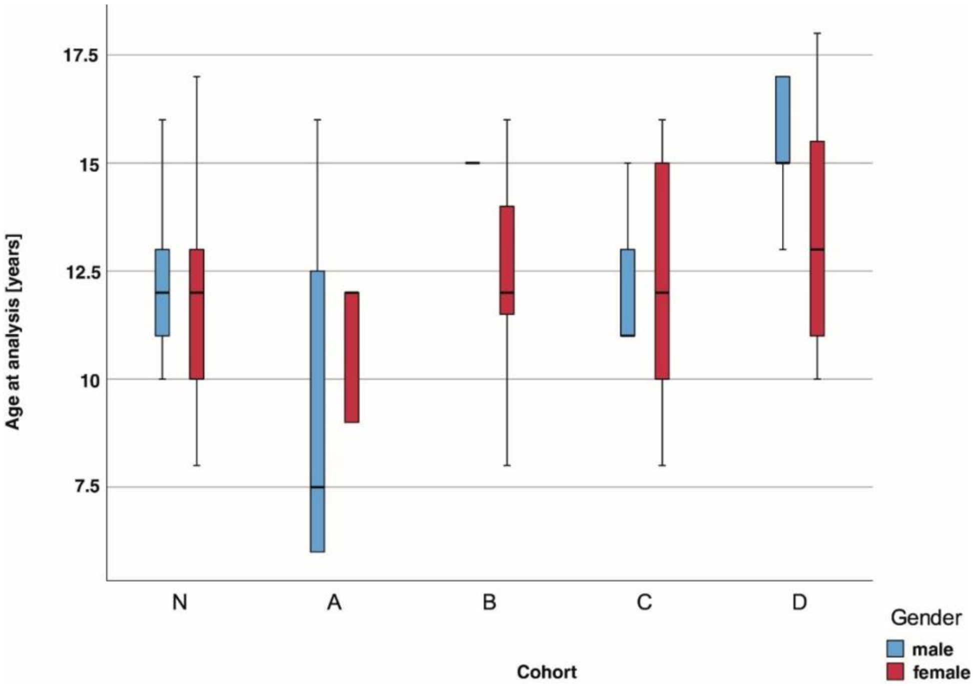

S2. Age-dependence of ODD location/ probands with superficial ODD are older than those with deep ODD. N; healthy controls. A, B, C, D, subgroups of the ODD population, respectively.
