# Supplementary material for: Retinal peripapillary nerve fiber and retinal ganglion cell layer thickening preceed atrophy in children and teenagers with optic disc drusen
Source: Sci Rep. 2025 Nov 7;15:39001. doi: 10.1038/s41598-025-25161-7 (PMC12595069; doi:10.1038/s41598-025-25161-7)
Supplement: Supplementary file 3 — Supplementary Material 3 [file 41598_2025_25161_MOESM3_ESM.pdf]

Figure S3.

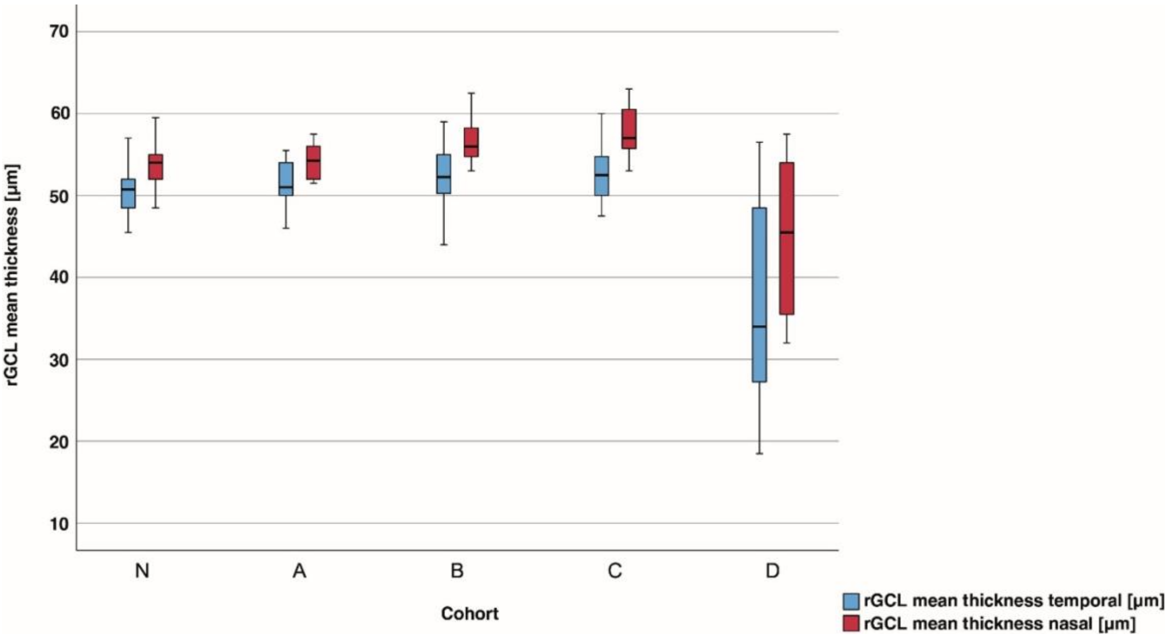

S3. Naso-temporal asymmetry of the rGCL. The decrease in rGCL thickness is larger temporally than nasally. The naso-temporal difference is significantly higher in group D versus controls ($p<0,001$, Mann Whitney Test). N; healthy controls. A, B, C, D, subgroups of the ODD population, respectively.
