# Supplementary material for: Retinal peripapillary nerve fiber and retinal ganglion cell layer thickening preceed atrophy in children and teenagers with optic disc drusen
Source: Sci Rep. 2025 Nov 7;15:39001. doi: 10.1038/s41598-025-25161-7 (PMC12595069; doi:10.1038/s41598-025-25161-7)
Supplement: Supplementary file 4 — Supplementary Material 4 [file 41598_2025_25161_MOESM4_ESM.pdf]

Table S1 Sensitivity analysis for the right and left eyes of ODD patients

	Right eyes	Left eyes
pRNFL 3.5 mm global	PC=-0.118<br><br>p=0.535	PC=-0.268<br><br>p=0.159
pRNFL 4.7 mm global	PC= -0.399<br><br>p=0.029	PC=-0.432<br><br>p=0.019
rGCL global	PC=-0.473<br><br>p=0.008	PC=-0.445<br><br>p=0.015

Table S1: Sensitivity analysis, carried out separately for the right and left eyes of ODD patients, showes that the effects for the central results rGCL global and pRNFL global for the 3.5 and for the 4.7 mm ring are stable. PC pearson correlation, p indicates the significance of the correlation.
